# Pharyngolaryngeal spasm‐induced dysphagia in an epileptic patient undergoing vagus nerve stimulation therapy

**DOI:** 10.1002/ccr3.2761

**Published:** 2020-02-26

**Authors:** Luca Castellani, Valentina Chiesa, Alberto Maccari, Emanuela Fuccillo, Maria Paola Canevini, Giovanni Felisati, Alberto Maria Saibene

**Affiliations:** ^1^ Otolaryngology Unit ASST Santi Paolo e Carlo Department of Health Sciences Università degli Studi di Milano Milan Italy; ^2^ Regional Centre for Epilepsy ASST Santi Paolo e Carlo Department of Health Sciences Università degli Studi di Milano Milan Italy; ^3^ Institute of Otorhinolaryngology Department of Clinical Sciences and Translation Medicine Università di Roma Tor Vergata Rome Italy

**Keywords:** drug‐resistant epilepsy, hypopharynx torsion, laryngeal electromyography, vagus nerve stimulation, vocal cord palsy

## Abstract

Vagus nerve stimulation for refractory epilepsy may induce laryngeal side effects such as dysphonia and dysphagia. Careful tuning of the stimulation parameters and collaboration between epileptologists and otolaryngologists can help significantly reduce side effects.

## Graphical abstract

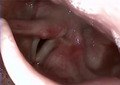

## 1 INTRODUCTION

According to the International League Against Epilepsy (ILAE), Drug‐Resistant Epilepsy (DRE) may be defined as failure of adequate trials of two tolerated and appropriately chosen and used antiepileptic drug (AED) schedules (whether as monotherapies or in combination) to achieve sustained seizure freedom.^1^ Currently, about 30% of seizure patient do not obtain an adequate control of symptoms despite a correct AED therapy.^2^ In these cases, nonpharmacological options consist in epilepsy surgery and, whether not suitable, vagus nerve stimulator (VNS) implantation. VNS therapy was approved in 1997 by the US Food and Drug Administration for patients older than 12 years of age affected by intractable partial seizures,^3^ and it is constituted by an electrode catheter which takes contact with the left vagus nerve (VN) to grant cyclic electrical burst to the left VN. During a well‐consolidated and low‐risk surgical procedure,^4^ the electrode is connected by an electrical wire to a chest‐implantable impulses generator, with an external control that allows to regulate burst intensity, frequency and duration in order to obtain the best seizure control and side effects reduction.

Side effects of VNS therapy could be due to the surgery itself (early complications) or to the stimulation of VN (late complications). Early complications consist in intraoperative bradycardia (1/1000 cases), infections (3%‐8%), and VN injury. Late complications consist mainly in laryngeal dysfunction with hoarseness, dyspnoea and cough, usually related to the frequency of stimulation,^5^ vocal fold palsy,^6^ laryngopharyngeal reflux,^7^ and obstructive sleep breathing disorders.^8^ While most of these alterations have been related to the variation of vocal cord movements during stimulation or to the orthodromic effect of VN stimulation, no study associated, at present, abnormal laryngopharyngeal movements with dysphagia symptoms, which are nevertheless well‐known in VNS patients.

We report the case of a 33‐year‐old man affected by drug‐resistant focal cryptogenic epilepsy who underwent VNS implantation and developed progressive invalidating dysphagia over the course of 6 months after successful implantation. Given the overall good response in terms seizure reduction, we strove to avoid VNS removal in this patient. The collaboration of otolaryngologists and epileptologists allowed for side effects control through careful tuning of stimulation parameters (amplitude of pulse current, stimulation frequency, and pulse width) during fiber‐optic evaluation.

## 2 CASE PRESENTATION

A 33‐year‐old man, affected by focal cryptogenic epilepsy since the age of 22 with no comorbidity, no developmental delay, and normal cognitive status, came to our observation with a diagnosis of refractory epilepsy. He had been previously treated in our centre with AED schedules with gradually poorer control of seizures, and he had been referred to focal epilepsy surgery with no improvements. He was therefore prescribed VNS implantation by our epileptologists.

The intervention was performed under general anesthesia, and the intra‐ and postoperative course was uneventful. The VNS was activated, as usual, 2 weeks after implantation, starting with low stimulation parameters, gradually increased in the following visits to reach the “maximum tolerable level,” that is, the highest intensity setting not inducing side effects such as cough or throat pain. After undergoing VNS therapy with therapeutic settings (intensity of 1.75 mA, stimulation frequency of 20 Hz, and single stimulation duration of 250 ms) for nearly 3 months, the patient presented with invalidating dysphagia, dysphonia, and cough during the active stimulation phase, which disappeared during VNS deactivation.

## 3 CLINICAL INVESTIGATION

### 3.1 Endoscopic laryngeal examination

**Figure 1:**
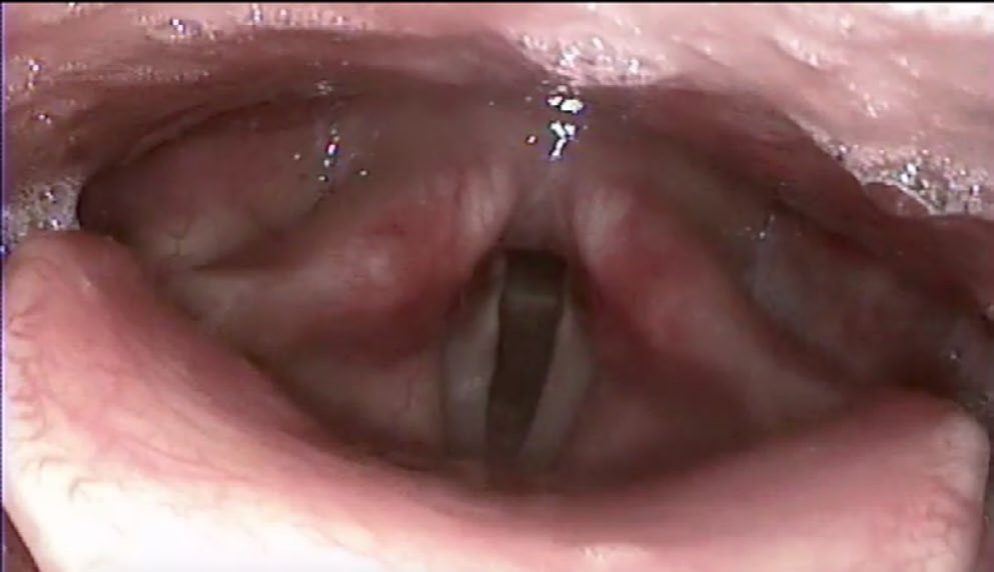
The image shows the endoscopic evaluation of the upper aerodigestive tract during the VNS inactivation. No alteration can be seen, with normal, symmetrical appearance of the structures

**Figure 2:**
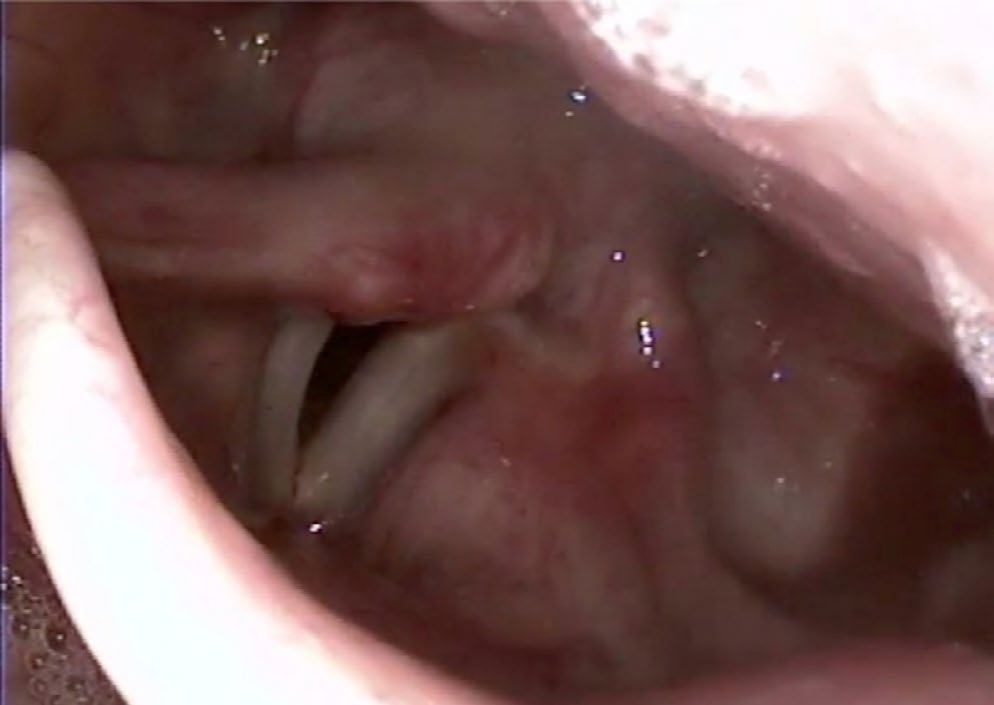
The image shows the endoscopic evaluation of the upper aerodigestive tract during the VNS activation with baseline parameters (intensity of 1.75 mA, stimulation frequency of 20 Hz, and single stimulation duration of 250 ms). The left vocal cord is adducted and a 30‐degree torsion of the left emi‐hypopharinx and epiglottis can be observed

The patient underwent an awake endoscopic laryngeal examination with flexible endoscopy, and the video was recorded on a portable device to better analyze the images with a slow‐motion feature. While the examination was completely normal during the VNS inactive phase (see Figure 1), during VNS activation, the patient referred all the aforementioned symptoms, and we identified an adduction of left vocal cord (VNS laryngeal pattern Group II according to Felisati classification^6^). The vocal cord adduction was coupled with a 30‐degree torsion of the left emi‐hypopharinx and epiglottis (see Figure 2).

### 3.2 Laryngeal muscles electromyography

In order to better explain the physiological bases of such alterations, the activity of thyroarytenoid (TA), posterior cricoarytenoid (PCA), and cricopharyngeal (CP) muscles were studied by a dual channel laryngeal electromyography (LMEMG), as already done by our group.^9^ LMEMG is an easy and safe procedure, performed under local anesthesia, that allows selective study of single muscles. The activity of the thyroarytenoid muscle is evaluated by insertion of a needle electrode at the level of cricothyroid space, just below the inferior margin of the thyroid cartilage. The activation of the thyroarytenoid (TA) muscle, hence the correct positioning of electrode, is verified by asking the patient to pronounce a sustained “I.” The needle for the posterior cricoarytenoid (PCA) muscle is inserted posteriorly to the thyroid lamina and the correct position checked by asking the patient to sniff. Swallowing is the selected task for checking the correct position of the cricopharyngeal (CP) needle.

The examination was performed both at rest and during activation of VNS, recording bilateral activity of muscles.

The results showed a normal neuromuscular activity in all the right side muscles examined and normal motor unit parameters (MUP) for all the muscles. A floating tonic activity, during normal breathing and OFF‐phase, of PCA left muscle was identified, with a further enhancement of this activity during ON‐phase (spasmogenic trend). As for the CP left muscle, a similar basal tonic activity during both OFF and ON‐phase was recorded, although with a complete pause of muscular activity during OFF‐phase and a fragmentation and irregular pause during ON‐phase.

These results confirmed an involvement of the upper esophageal sphincter involuntary musculature, thus giving a solid explication of the clinical manifestations.

## 4 TREATMENT, OUTCOME, AND FOLLOW‐UP

In order to mediate the clinical manifestation of side effects with a good seizure control, in collaboration with our neurologist team, we tried to modify the parameters of VNS looking for those which guaranteed the best control of seizure and limited side effects. In order to optimize the outcome, such tuning was performed under fiber‐optic endoscopy (see Video S1).

With a slight decrease in overall parameters (1.00 mA, 20 Hz, 250 μs), the left VC remained adducted, while the epiglottis laid in its normal anatomical position, with a less pronounced emi‐hypopharinx torsion. Nevertheless, the patient still complained dysphagia, cough, and dysphonia.

Decreasing both intensity and frequency of stimulation at a value below the parameters recommended by the manufacturer (1.00 mA, 10 Hz, 250 μs), we obtained a paradoxical tremor of left hypopharynx, larynx, and epiglottis. The pathophysiological explication of this phenomenon could be an incomplete muscle tetanus resulting in myoclonus.

By reduction of time stimulation time, with intermediate frequency (1.00 mA, 20 Hz, 130 μs) the left VC adduction remained but without any spasm of the hypopharyngeal musculature. The patient referred no symptoms of dysphagia or dysphonia with this setting, which were then confirmed for its normal therapy. The patient then underwent regular controls after 3 months, 6 months, and 1 year reporting stable seizure control without dysphagia.

## 5 DISCUSSION

More than 20 years after its FDA approvation, VNS still maintains its therapeutic role in the treatment of refractory epilepsy; thanks to its high efficacy profile (in patients who otherwise would not have other equally effective therapeutic options) and good tolerability. Studies show a mean seizure frequency reduction by 26% after 1 year, 30% after 5 years, and 52% after 12 years with VNS treatment.^10^ In a recent Japanese study, seizure control improved over time with median seizure reduction of 25.0%, 40.9%, 53.3%, 60.0%, and 66.2% and responder rates of 38.9%, 46.8%, 55.8%, 57.7%, and 58.8% at 3, 6, 12, 24, and 36 months of VNS therapy, respectively.^11^

Despite its efficacy and safety, the VNS implant could present a group of adverse effects (AE) the causes of which remain to be more thoroughly investigated. Common AE linked with VNS activation are hoarseness (1.4%‐64%), voice alterations (6%‐66%), dyspnea (2%‐25%), throat pain (4.7%‐22%), cough (7%‐45%), neck pain and/or tingling and twitching in the neck muscles (0.5%‐22%), dysphagia (13%‐17.9%), headache (7%‐30%), and chest pain (up to 13%).^13^ Most of the side effects of VNS are recorded during the on‐phase of the stimulation and seem to be dose‐dependent, making it possible to reduce them through the fine adjustment of the VNS parameters.^12^

We are not yet able to explain the exact pathophysiological mechanisms underlying all VNS side effects, which could be due to orthodromic activation of the recurrent laryngeal nerve, antidromic activation, or both. We also identified a certain number of patients with the presence of spasms of the left vocal cord during the OFF‐phase of the VNS which cannot yet be traced back with certainty to an iatrogenic cause occurred at the time of implantation or to a progressive damage following nerve stimulation.^9^ However, our studies showed that the laryngeal effects induced by the VNS are not dependent on the positioning of the electrode in relation to the position of the recurrent laryngeal nerve or the superior laryngeal nerve.

To our knowledge, this is the first paper which investigates the dysphagia as a side effect after VNS implantation and tries to find a pathophysiological cause other than laryngopharyngeal reflux.^7^ A 2018 case report 14 describes a patient who suffered pain and shock‐like sensation during rotation of neck to the right. In this case, the VNS electronic interrogation returned an increased impedance when the patient turned his head to the right, with consequent induced pain. In this case, the VNS was removed, and during the removal procedure, a wire lead fracture was identified, which presumably led to an electrical discharge in the neck. Previous studies recorded cases of inhalation during activation of VNS. In Ref.^15^ the authors advocate a possible action of the vagus nerve stimulation on pharyngoesophageal reflexes, leading to a relaxation of the upper part of the esophagus during the act of swallowing or an altered neuronal activity in the solitary tract and ambiguous nuclei of the brainstem, in children with severe mental and motor impairment. In another study,^16^ the authors did not identify any direct effect of VNS implantation in patients with aspiration during VNS stimulation, though they observed a transient left vocal cord paralysis, which did not appear to have any correlation to the swallowing dysfunction.

Our investigations, instead, found an anatomical and physiological cause explaining the symptom dysphagia after implantation of VNS, and thus, we were able to identify a direct connection between VNS activation and recruitment of motor units of the pharyngolaryngeal muscle fibers.

Despite the observations and parameters, tuning we performed in this specific patient cannot be generalized to all VNS patients referring dysphagia and dysphonia; this case report highlights once more how the multidisciplinary evaluation of these patients with the use of flexible endoscopy is mandatory in the management of VNS side effects. Furthermore, our findings demonstrated how important is to fine‐tune VNS parameters, in order to minimize side effects while obtaining the best seizure control.

## 6 CONCLUSIONS

VNS implantation is a valid and safe therapeutic option for the treatment of refractory epilepsy. The adverse effects, although not yet fully explained, are known and can be modulated by adjusting the stimulation parameters of the pulse generator. Our experience shows that the close collaboration between ENT surgeons and epileptologists, able to combine the anatomical knowledge of the cervical region and endoscopic access to the side effects site with the electrophysiological knowledge of the VNS, is essential for the reaching of this goal. Surgery for complete removal or revision and replacement of the device should be regarded as an extreme measure, to be considered only in cases of device malfunction (4%‐16.8%), failure of VNS therapy, intolerable side effects, or patients' specific requests.^5^

## CONFLICT OF INTEREST

None declared.

## AUTHOR CONTRIBUTIONS

LC and EF: wrote the article; AM and GF: performed surgery for VNS implantation; VC and AMS: performed the endoscopic study and the stimulation tuning; MPC: performed the epileptological follow‐up of the patient.

## Supporting information

VideoS1

## References

[1] Kwan P , Arzimanoglou A , Berg AT , et al. Definition of drug resistant epilepsy: Consensus proposal by the ad hoc Task Force of the ILAE Commission on Therapeutic Strategies. Epilepsia. 2010;51:1069‐1077. 19889013 10.1111/j.1528-1167.2009.02397.x

[2] Brodie MJ . Road to refractory epilepsy: the Glasgow story. Epilepsia. 2013;54:5‐8. 10.1111/epi.12175 23646962

[3] Cyberonics, Inc . VNS therapy products manuals and safety alerts: Part I ‐ Introduction ‐ Indications, Warnings, and Precautions, p. 7–13. http://dynamic.cyberonics.com/manuals/. Accessed October 1, 2012.

[4] Felisati G , Saibene AM , Canevini MP . In reference to treatment of epilepsy by stimulation of the vagus nerve from head‐and‐neck surgical point of view. Laryngoscope. 2015;125(9):E326. 25641055 10.1002/lary.25131

[5] Giordano F , Zicca A , Barba C , Guerrini R , Genitori L . Vagus nerve stimulation: surgical technique of implantation and revision and related morbidity. Epilepsia. 2017;58:85‐90. 10.1111/epi.13678 28386925

[6] Felisati G , Gardella E , Schiavo P , et al. Endoscopic laryngeal patterns in vagus nerve stimulation therapy for drug‐resistant epilepsy. Eur Arch Otorhinolaryngol. 2014;271:117. 23744179 10.1007/s00405-013-2568-z

[7] Marciante GA , Felisati G , Pipolo C , et al. Vagus nerve stimulation therapy induces laryngopharyngeal reflux: a preliminary evaluation. B‐ENT. 2017;13:275‐279.

[8] Zambrelli E , Saibene AM , Furia F , et al. Laryngeal motility alteration: a missing link between sleep apnea and vagus nerve stimulation for epilepsy. Epilepsia. 2016;57:e24‐e27. 26589721 10.1111/epi.13252

[9] Saibene AM , Zambrelli E , Pipolo C , et al. The role of laryngeal electromyography in vagus nerve stimulation‐related vocal fold dysmotility. Eur Arch Otorhinolaryngol. 2017;274:1585. 27738822 10.1007/s00405-016-4344-3

[10] Uthman BM , Reichl AM , Dean JC , et al. Effectiveness of vagus nerve stimulation in epilepsy patients. Neurology. 2004;63(6):1124‐1126. 15452317 10.1212/01.wnl.0000138499.87068.c0

[11] Kawai K , Tanaka T , Baba H , et al. Outcome of vagus nerve stimulation for drug‐resistant epilepsy: the first three years of a prospective Japanese registry. Epileptic Disord. 2017;19(3):327‐338. 28832004 10.1684/epd.2017.0929

[12] Wheless JW , Gienapp AJ , Ryvlin P . Vagus nerve stimulation (VNS) therapy update. Epilepsy Behav. 2018;88:2‐10. 10.1016/j.yebeh.2018.06.032 30017839

[13] Timarova G , Šteňo A . Late‐onset jaw and teeth pain mimicking trigeminal neuralgia associated with chronic vagal nerve stimulation: case series and review of the literature. BMC Neurol. 2017;17(1):113. 28619068 10.1186/s12883-017-0892-4 PMC5473002

[14] D'Agostino E , Makler V , Bauer DF . Vagal nerve stimulator malfunction with change in neck position: case report and literature review. World Neurosurg. 2018;114:165‐167. 29555606 10.1016/j.wneu.2018.03.073

[15] Lundgren J , Ekberg O , Olsson R . Aspiration: a potential complication to vagus nerve stimulation. Epilepsia. 1998;39:998‐1000. 9738680 10.1111/j.1528-1157.1998.tb01450.x

[16] Schallert G , Foster J , Lindquist N , Murphy JV . Chronic stimulation of the left vagal nerve in children: effect on swallowing. Epilepsia. 1998;39:1113‐1114. 9776333 10.1111/j.1528-1157.1998.tb01298.x

